# Unraveling the Relationship Between English Learning Burnout and Academic Achievement: The Mediating Role of English Learning Resilience

**DOI:** 10.3390/bs14121124

**Published:** 2024-11-24

**Authors:** Honggang Liu, Ling Jin, Xiaoyu Han, Haoyue Wang

**Affiliations:** 1 School of Foreign Languages, Soochow University, Suzhou 215006, China; liuhonggang@suda.edu.cn; 2 School of Foreign Languages, Guilin Tourism University, Guilin 541006, China; 3 School of Foreign Languages, Northeast Normal University, Changchun 130024, China; 4 School of Foreign Languages, Boda College of Jilin Normal University, Siping 136000, China; wdaisy2023@163.com

**Keywords:** English learning burnout, English learning resilience, academic achievement, mediation analysis, senior high school students

## Abstract

Although burgeoning research has been conducted on the role of negative emotions (e.g., English learning burnout) in affecting students’ academic achievement, there are limited studies on the intricate working mechanism between these two factors. Academic resilience is an adaptive response to academic adversity and might therefore offer protection against negative emotions (e.g., English learning burnout). Hence, this study focused on the complex interplay among students’ English learning burnout, English learning resilience, and academic achievement. A total of 334 senior high school students were recruited in the current study. The findings displayed that students’ English learning resilience mediated the relationship between English learning burnout and English academic achievement. This study may generate suggestions and implications for English teaching and learning.

## 1. Introduction

Burnout, initially identified as an occupational hazard, describes a psychological syndrome emerging as a prolonged response to chronic workplace stressors [1]. This concept, involving overwhelming exhaustion, feelings of cynicism, and a sense of inefficacy [1], has recently been introduced into the domain of foreign language education [2,3]. The process of language acquisition demands extensive memorization, imitation, and practice. Given the inherent challenges of language learning, English-as-a-foreign-language (EFL) learners are at risk of experiencing burnout related to English learning. For Chinese EFL learners, the demands are particularly heightened, as English has been a compulsory subject within the curriculum since primary school, with scores playing a pivotal role in determining admission to middle schools and universities. In such a context, where English learning may be perceived as an obligatory task with limited autonomy, students’ interest in English learning may be eroded. Consequently, they may view English learning as a “burden”, increasing their susceptibility to burnout [3].

Furthermore, current studies have highlighted the role of burnout in students’ learning psychology and behaviors. Individuals with higher levels of academic burnout tend to have higher dropout rates [4], lower subjective well-being [5], and poor mental health [6,7]. English learning burnout, as a multifaceted construct, may exist as a great challenge and adversity for students, affecting their educational outcomes. English learning resilience, a crucial protective factor, enables students to effectively manage the challenges encountered in academic learning and recover from adverse situations in the learning environment [8,9]. Theoretically, academic resilience is an adaptive response to academic adversity that promotes student learning and prevents negative psychological syndromes (e.g., burnout) from undermining students’ academic achievement. However, little research has been conducted on the intricate relationships between English learning burnout and academic achievement, as well as the mediating role of English learning resilience between them. To fill these voids, this study aims to explore the mediating effect of English learning resilience on the relationship between English learning burnout and academic achievement.

## 2. Literature Review

### 2.1. Understanding English Learning Burnout

Burnout refers to a complex psychological syndrome caused by chronic stressors [1]. Recent years have witnessed increasing attention on this topic in the field of foreign language education. Being introduced to this field, English learning burnout refers to students’ emotional and physical fatigue and decreased passion and self-confidence in language learning [2]. Previous studies have been focused on the exploration of the inner structure of English learning burnout. Some studies treated it as a tri-factorial construct comprising exhaustion (i.e., the feeling of fatigue English learners experience in English learning activities), cynicism (i.e., a detached and indifferent attitude toward English learning), and reduced efficacy (i.e., a decline in learners’ evaluation of their efficiency and ability to learn English) [3,10,11,12,13]. English learning burnout is a language-specific and context-related concept that may manifest in a manner consistent in the context of English learning. Liu and Zhong [2] conducted further in-depth research on the inner structure of English learning burnout. They investigated the inner structure of English learning burnout based on 1213 Chinese senior high school students, proving that English learning burnout is a bidimensional construct consisting of demotivation and exhaustion. This finding also verified that burnout as a multidimensional psychological variable displays a language-specific and context-related nature, which may contribute to the further in-depth investigation of English learning burnout.

In addition to research on the constructs of students’ English learning burnout, there have been limited attempts to explore the relationships between English learning burnout and English learning resilience. Existing studies have revealed a significant correlation between English learning resilience and burnout [14]. Researchers have also revealed that students’ English learning resilience can be affected by English learning burnout [15]. Moreover, the detrimental effect of English learning burnout has been revealed, indicating that students’ academic engagement can be negatively affected by their burnout in English learning [14]. In addition, researchers have attempted to explore the predictive factors of English learning burnout, suggesting that English teacher support [16], personality traits [17,18], growth language mindset [19], and coping styles [20,21] may affect English learning burnout.

### 2.2. Understanding English Learning Resilience

Resilience refers to “the process of, capacity for, or outcome of successful adaptation despite challenging or threatening circumstances” [22], students’ ability to effectively cope with academic setbacks and maintain good academic performance in the learning process [23,24], or students’ cognitive, affective, and behavioral reactions to encountering academic adversities [8]. English learning is a socialized process [25,26,27]. During the process of language learning, students face various difficulties. Students can actively explore their advantages and overcome all the difficulties encountered in learning to achieve satisfactory results, which will result in a virtuous cycle of learning. Protective factors can buffer or guard against the negative impact of risk factors, thereby helping students obtain further development. Lereya et al. [28] proposed a ten-factorial structure, including family connection, school connection, community connection, participation in home and school life, participation in community life, self-esteem, empathy, problem-solving, goals and aspirations, and peer support. Academic resilience has been revealed to be a multidimensional structure; for instance, Martin and Marsh’s [9] 5-C model (confidence, coordination, control, composure, and commitment), Kim and Kim’s [29] five-factorial structure, consisting of perceived happiness, empathy, sociability, persistence, and self-regulation, and Lereya et al.’s [28] ten-factorial structure.

Inspired by definitions of resilience in general education, resilience in language learning can be viewed as the process, ability, or production of successful adaptation in response to academic frustrations, difficulties, and the stress of learning a language [30,31]. Although some related research has been carried out, few studies have examined academic resilience in English learning [31,32]. Liu and Han [33] and Wei et al. [34] adopted Kim and Kim’s [29] five-factorial framework to investigate the structure of academic resilience among students in the Chinese context. Language learners in school are likely to encounter a multitude of challenges and pressures [32]. When facing academic adversity, students not only cultivate positive individual characteristics (e.g., self-esteem [29]) but also draw on support from significant others, such as teachers and parents [35]. Despite the limited number of studies on resilience in language learning, some studies have directly examined the structure of resilience found in psychology or general education. During the process of learning, students face various difficulties, inspired by Lereya et al.’s [28] structure. Duan et al. [32] validated a four-factorial structure of student English learning resilience, including positive individual characteristics, family support, teacher support, and peer support.

To date, few studies have investigated academic resilience in the language learning context [33,34]. As a complex learning subject, other psychological factors of students in the process of foreign language learning are the formation and development of academic resilience, which is closely related to positive psychological factors such as mindfulness, happiness, perseverance, learning commitment, and language self-confidence, as well as negative psychological factors, such as school aversion and anxiety sensitivity. Generally, academic resilience is positively correlated with positive psychological factors and negatively correlated with negative emotions. There is also a significant positive correlation between Chinese English learners’ positive psychological emotions (such as self-efficacy, motivation, and perseverance) and academic resilience [33,36]. Many scholars have discussed the relationship between academic resilience and academic achievement and found that academic resilience has a positive predictive effect on academic achievement [33,37,38].

### 2.3. Hypothetical Model of the Relationship Between Resilience, Burnout, and Academic Achievement

English learning resilience and burnout, as vital positive psychological factors and negative emotional factors, respectively, are strongly related to English achievement and performance. Many scholars have discussed the positive relationship between academic resilience and academic achievement [37,38]. Resilience is generally seen as the effective recovery and adaptation of the individual in a dilemma, thus achieving sound development, the essence of which lies in positive adaptation and adjustment in a state of an unfavorable environment [39]. The relationship between resilience and educational success has been reported to be positive [40]. Academically resilient students are capable of sustaining higher levels of academic achievement, engagement, and consistent class attendance despite the presence of stressful events and moods (e.g., academic burnout) [9]. Language learning is a sustained and often tedious process [33], and students inevitably encounter setbacks or difficulties such as poor exam marks. Faced with such stressors, some students adapt positively and develop well, but others struggle with maladjustment problems, such as academic burnout, leading to further decline in the student’s academic performance. However, few studies have investigated the intricate mechanism between English learning burnout and academic achievement, and the potential mediating effect of English learning resilience remains uncertain. To address these gaps, the present study aimed to explore the relationships among students’ English learning burnout, English learning resilience, and academic achievement.

**Figure 1:**
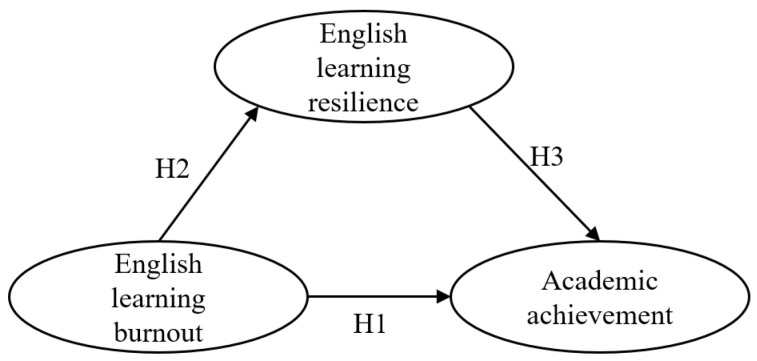
Hypothetical model of the relationship between English learning burnout, resilience, and academic achievement.

To be more precise, the following hypothesized model (see Figure 1) was proposed:

The hypotheses are as follows:

**Hypothesis** **1.** *English learning burnout could directly impact academic achievement.*

**Hypothesis** **2.** *English learning burnout could directly impact English learning resilience.*

**Hypothesis** **3.** *English learning resilience could have a direct impact on academic achievement.*

**Hypothesis** **4.** *English learning burnout could indirectly impact academic achievement via the mediation of English learning resilience.*

## 3. Method

### 3.1. Participants

A total of 365 senior high school students from a city in Northeast China were recruited for this survey via random sampling because of its practical advantages in ensuring accessibility [41]. English is a compulsory subject within their curriculum. They have learned English as a foreign language for 6 to 8 years. The participants were invited to provide responses regarding their experiences of burnout and resilience within the English learning context. Prior to participation, participants received comprehensive briefings on the research objectives, with assurances of anonymity and confidentiality provided. Following a rigorous screening process, a valid sample of 334 students was obtained, with invalid responses excluded from analysis. Among this cohort, 130 students (38.9%) were male, while 204 (61.1%) were female. The age of the participants ranged from 15 to 18 years old, with the mean age being 16.31 years old (*SD* = 0.86).

### 3.2. Instruments

The current study utilized a questionnaire as the instrument to collect quantitative data. The questionnaire starts with questions about respondents’ personal information, such as gender and grade. The subsequent sections used two 6-point Likert scales about burnout and resilience. All the items on the two scales were rated from 1 (“strongly disagree”) to 6 (“strongly agree”). To ensure participants’ clear comprehension of all the items, the English versions of the scales were translated into Chinese through the following procedures. The first author undertook the initial translation. Subsequently, the translated scale was scrutinized by a Chinese–English bilingual individual. Any minor discrepancies that arose between the two translators were resolved through discussion.

#### 3.2.1. English Learning Burnout

The Senior High School English Learning Burnout Scale [2], which has two dimensions, exhaustion (4 items) and demotivation (6 items), was used to measure students’ English learning burnout. The sample item for exhaustion was “Studying or attending an English class is really a strain for me” and the sample item for demotivation was “I believe that I don’t make an effective contribution to the English classes that I attend”. The reliability for exhaustion, demotivation, and the whole scale was satisfactory, with Cronbach’s α values being 0.949, 0.914, and 0.943, respectively. The scale also had acceptable structural validity (*χ*^2^/*df* = 2.371, RMSEA = 0.064, SRMR = 0.042, CFI = 0.987, TLI = 0.981).

#### 3.2.2. English Learning Resilience

English learning resilience was measured by the Student Academic Resilience in English Learning Scale [32], which involves four dimensions, namely positive individual characteristics (8 items), teacher support (5 items), peer support (4 items), and family support (4 items). The sample items for positive individual characteristics, teacher support, peer support, and family support were “I have goals and plans for future English learning”, “The English teacher is very concerned about my study”, “My classmates would point out my mistakes in English learning and encourage me”, and “My parents assist me in learning English”. This 21-item scale was found to have sound reliability (Cronbach’s α = 0.911). Cronbach’s α values of positive individual characteristics, teacher support, peer support, and family support were 0.917, 0.791, 0.880, and 0.724, respectively. The structural validity was reasonable, with *χ*^2^/*df* = 2.159, RMSEA = 0.059, SRMR = 0.054, CFI = 0.968, and TLI = 0.958.

#### 3.2.3. Academic Achievement

Students’ academic achievement was measured by their scores on a final English exam with 150 full marks, the unified examination in the whole city. This exam was designed to emulate Gaokao, China’s highly competitive and influential college entrance examination that determines university admission and significantly shapes students’ future trajectories. The English exam, mirroring Gaokao’s structure, includes question types such as listening, reading comprehension, a cloze test, grammar filling, and writing. To assess their performance, students were asked to fill in their scores from this exam in an online questionnaire. This approach provides a standardized metric for evaluating English proficiency within our study’s context.

### 3.3. Data Collection and Analysis

The electronic questionnaire was disseminated among Chinese high school English learners through an online platform known as Wenjuanxing (https://www.wjx.cn/; accessed on 20 May 2023). Some English teachers were invited to share the questionnaire with their students via social media platforms, including WeChat and QQ. Prior to participation, the objectives of the research were thoroughly explained to both the participants and their guardians, who were assured of the anonymity and confidentiality of their responses. Altogether, 365 responses were obtained. After eliminating invalid responses, 334 valid responses were retained. SPSS 26.0 and AMOS 24.0 were then used to analyze the data.

With an overview of the data based on descriptive and correlation analyses, a test of a hypothesized simple structural equation model (SEM) was applied to examine the direct and indirect effects of English learning burnout on academic achievement via English learning resilience. A complex SEM was also used to examine the effect of English learning burnout on academic achievement via four dimensions of English learning resilience (i.e., positive individual characteristics, teacher support, peer support, and family support). The significance of the mediating effect attained using the bootstrapping approach yields a bias-corrected 95% confidence interval by taking repeated samples (5000 bootstrap samples) [42]. The mediating effect was significant if the confidence interval did not straddle zero [43]. To evaluate how well the empirical data fit the hypothesized model, the following indices were used: *χ*^2^*/df* should be less than 5, the CFI and TLI should exceed 0.90, and the RMSEA and SRMR should be below 0.08 [44].

## 4. Results

### 4.1. Profiles of Burnout and Resilience in Students’ English Learning

Table 1 shows that Chinese senior high school students experienced low levels of English learning burnout (*M* = 2.29; *SD* = 1.26), exhaustion (*M* = 1.98; *SD* = 1.35), and demotivation (*M* = 2.49; *SD* = 1.36). They reported a moderate-to-high level of English learning resilience (*M* = 4.77; *SD* = 0.91), with high degrees of teacher support (*M* = 5.38; *SD* = 0.80) and moderate-to-high levels of peer support (*M* =4.68; *SD* = 1.31), positive individual characteristics (*M* = 4.30; *SD* = 1.29), and family support (*M* = 4.82; *SD* = 1.12). In addition, the variables were significantly correlated with each other but to different degrees.

### 4.2. Relationship Among English Learning Burnout, Resilience, and Academic Achievement

**Figure 2:**
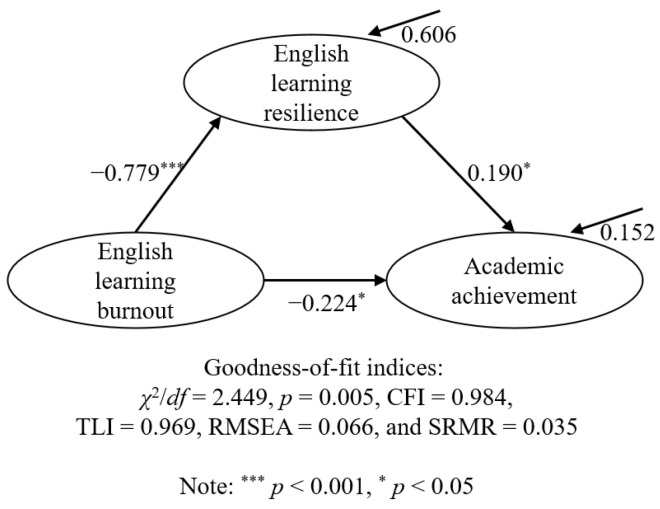
The final model of the relationship between English learning burnout, English learning resilience, and academic achievement.

The SEM demonstrated good model fit indices, with RMSEA and SRMR values of 0.066 and 0.035, respectively, and CFI and TLI values of 0.984 and 0.969, respectively. As shown in Figure 2, the results of the SEM indicated that senior high school students’ English learning burnout could negatively affect their academic achievement in a direct manner (*β* = −0.224; *p* < 0.05), confirming Hypothesis 1. In contrast, English learning resilience had a positive effect on academic achievement (*β* = 0.190; *p* < 0.05), supporting Hypothesis 3. Moreover, English learning resilience was negatively predicted by English learning burnout (*β* = −0.779; *p* < 0.001). Thus, Hypothesis 2 was verified. The results of the mediation analysis indicated that English learning resilience could act as a mediator in the link between English learning burnout and academic achievement (*β* = −0.148). Hence, Hypothesis 4 was accepted.

**Figure 3:**
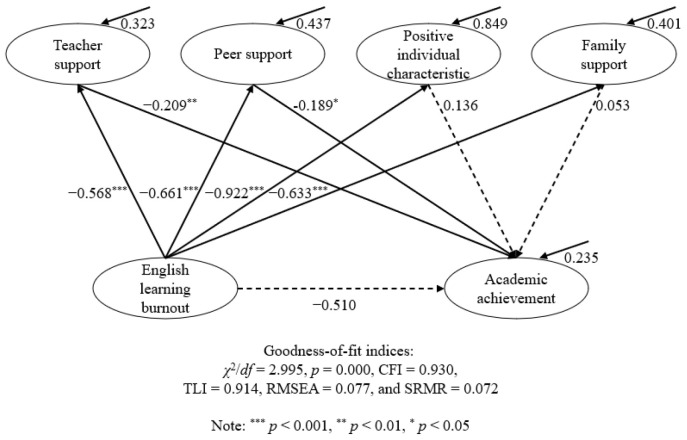
The final model of the mediation of the dimensions of resilience between burnout and academic achievement.

To delve into the specific functions of the four dimensions of teacher resilience, the model shown in Figure 3 was established.

As shown in Table 2, English learning burnout could not directly predict academic achievement (*β* = −0.510; *p* = 0.079). English learning burnout could also predict four dimensions of English learning resilience, namely positive individual characteristics (*β* = −0.992; *p* < 0.001), teacher support (*β* = −0.568; *p* < 0.001), peer support (*β* = −0.661; *p* < 0.001), and family support (*β* = −0.633; *p* < 0.001). Academic achievement could be positively predicted by two dimensions of English learning resilience, namely teacher support (*β* = −0.209; *p* < 0.01) and peer support (*β* = −0.189; *p* < 0.05). Regarding the mediating effect of the dimensions of English learning resilience, teacher support and peer support could mediate the link between English learning burnout and academic achievement (*β* = 0.119, *p* < 0.01; *β* = 0.125; *p* < 0.05).

## 5. Discussion

This study revealed that Chinese senior high school students experienced a low level of English learning burnout, which echoes the results of prior studies [2,3,45]. This low level may imply that senior high school students may adopt behavioral adjustment strategies [46] and apply emotional and cognitive adjustment strategies [47] to self-regulate the stress caused by English learning difficulties, thereby reducing burnout. Chinese senior high school students had a relatively high level of English learning resilience, which has been evidenced in previous studies [30,33]. It indicated that senior high school students possessed some capacity that enabled them to recover from academic adversities, adapt, thrive in their studies, and promote academic achievement through learning activities. Furthermore, this study revealed a weak negative correlation between English learning burnout and academic achievement, similar to prior studies [48]. Senior high school students were likely to take measures to seek behavioral and emotional strategies to cope with academic problems and decrease their exhaustion and demotivation in English learning. Moreover, senior high school students’ English learning burnout had a moderate negative correlation with English learning resilience. Students with a high level of English learning resilience will be full of confidence, maintain a positive attitude when they encounter setbacks and challenging situations, persist, concentrate on solving problems encountered, and take the initiative to seek family and other interpersonal relationships to vent their emotions (e.g., English learning burnout [49]).

The SEM showed that senior high school students’ English learning burnout significantly and negatively predicted resilience, which is consistent with previous studies revealing a significant association between these two variables [14,15]. Furthermore, the results of the current study offer valuable insights into the relatively scarce body of research concerning the impact of English learning burnout on resilience. The structural equation modeling (SEM) analysis revealed a significant negative correlation between high school students’ English learning burnout and resilience, aligning with prior research that has established a substantial link between these two variables [14,15]. In addition, this study revealed the indirect negative impact of English learning burnout on academic achievement through the mediating role of English learning resilience, which is reasonable since previous research has shown a negative association between English learning burnout and resilience [49] and a positive impact of English learning resilience on academic achievement [33].

This study further explored the mediating effect of four dimensions of English learning resilience on the relationship between English learning burnout and academic achievement. This may significantly enhance our grasp of how and which dimension of English learning resilience operates as a buffer in the relationship between burnout and academic success. Given that EFL learning occurs primarily in EFL classes and that language classes are typically characterized by frequent interactions and evaluations on the part of teachers, peers, and EFL learners [50], the role of teacher and peer support is more crucial than that of other sources. Evidence has shown that positive relationships between students and teachers, facilitated by EFL teachers’ consistent sympathy, recognition, and care, fulfill the emotional needs of learners and contribute to the cultivation of a positive classroom environment, which helps students avoid unfavorable academic outcomes such as burnout [51]. Studies have also shown that peer support similarly plays an essential role in the academic performance and well-being of EFL learners, given that learners spend a substantial amount of time with their classmates, who may often face similar academic challenges throughout the language learning process [52].

## 6. Conclusions and Implications

The current study revealed the predictive effect of English learning burnout on academic achievement, indicating the significance of and urgency for interventional procedures to help students alleviate or recover from English learning burnout. The mediating role of specific dimensions of English learning resilience was also investigated, contributing to a fuller and more comprehensive understanding of the functioning of English learning resilience in the links between English learning burnout and academic achievement. This finding highlights the significance of internal protective factors (i.e., positive individual characteristics) and external protective factors (i.e., family support) in buffering and blocking the negative effect of English learning burnout on academic achievement. In addition, this study also validated the stable four-dimensional construct of English learning resilience.

The results of the present study may provide some implications for English educational practice, particularly within the educational context where learners are easily prone to burnout due to the compulsory nature and high-pressure environment associated with EFL learning. The predictive effect of English learning burnout and resilience on academic achievement highlights the urgency to mitigate burnout and promote resilience. The evidence suggests that a combination of both individual and organizational interventions is likely to be most effective [53]. Individual interventions should focus on raising awareness about burnout and promoting resilience, while organizational interventions could involve reducing academic pressures such as exam stress and managing parental undue expectations [54]. In the process of English learning, teachers should not only provide students with high-quality academic support but also build harmonious teacher–student relationships. They should be attentive to students’ learning conditions and mental health, offering help, care, and support to assist students in overcoming English learning burnout and achieving academic success. Furthermore, to foster sustainable improvement in English learning, students must evolve into autonomous learners capable of self-regulating their learning processes. Autonomous learners should have a clear understanding of their objectives, the ability to self-evaluate accurately, recognize their strengths and weaknesses, and possess the resilience to quickly recover from academic setbacks.

Although the authors have tried to reliably explore the links between English learning burnout, English learning resilience, and academic achievement, the study is subject to the following limitations: First, the respondents were all senior high school students in Jilin Province, which may overlook regional differences in students’ English learning burnout, English learning resilience, and achievement. To obtain a more comprehensive understanding of Chinese English learners’ burnout and resilience, further research can expand the research scope to different regions. In addition, the current study adopted a quantitative research design, relying on questionnaires to collect data. Therefore, future studies may apply interviews, diaries, observations, and field notes to obtain a fuller and more objective profile of students’ psychological states. Moreover, this study utilized English scores obtained from final exams that are recognized for their credibility within the educational context and subjected these scores to a rigorous standardization process to enhance the study’s reliability and validity. While acknowledging the inherent limitations in how well such exam results can reflect true language proficiency, the study suggests that scores from well-established and widely accepted standardized assessments, such as the GaoKao, IELTS, and TOEFL, serve as a robust indicator of academic achievement.

## Figures and Tables

**Table 1:** Descriptive statistics and correlations among variables.

	*M*	*SD*	1	2	3	4	5	6	7	8
1. Demotivation	2.49	1.36	——							
2. Exhaustion	1.98	1.35	0.737 **	——						
3. ELB	2.29	1.26	0.958 **	0.900 **	——					
4. PositiveCh	4.30	1.29	−0.736 **	−0.535 **	−0.702 **	——				
5. TeacherS	5.38	0.80	−0.467 **	−0.335 **	−0.444 **	0.501 **	——			
6. PeerS	4.68	1.31	−0.466 **	−0.351 **	−0.450 **	0.612 **	0.491 **	——		
7. FamilyS	4.82	1.12	−0.413 **	−0.320 **	−0.402 **	0.530 **	0.335 **	0.401 **	——	
8. ELR	4.77	0.91	−0.699 **	−0.516 **	−0.669 **	0.904 **	0.701 **	0.797 **	0.695 **	——
9. AcademicA	93.13	23.53	−0.366 **	−0.358 **	−0.388 **	0.358 **	0.098	0.144 **	0.234 **	0.293 **

Note: *N* = 334; ** *p* < 0.01; ELB = English learning burnout; PositiveCh = positive individual characteristics; TeacherS = teacher support; PeerS = peer support; FamilyS = family support; ELR = English learning resilience; AcademicA = academic achievement.

**Table 2:** Different paths of variables in structural equation modeling.

Pathway	B	*β*	S.E.	C.R.	*p*	R^2^
ELB→PC	−1.022	−0.922	0.063	−16.335	***	0.849
ELB→TS	−0.141	−0.568	0.020	−7.232	***	0.323
ELB→PS	−0.673	−0.661	0.061	−11.086	***	0.437
ELB→FS	−0.669	−0.633	0.066	−10.179	***	0.401
ELB→AA	−0.434	−0.510	0.247	−1.759	0.079	0.235
PC→AA	0.105	0.136	0.180	0.583	0.560	
TS→AA	−0.716	−0.209	0.251	−2.857	**	
PS→AA	−0.158	−0.189	0.067	−2.367	*	
FS→AA	0.043	0.053	0.066	0.649	0.516	

Note: *** *p* < 0.001; ** *p* < 0.01; * *p* < 0.05. ELB = English learning burnout; PC = positive individual characteristic; TS = teacher support; PS = peer support; FS = family support; AA = academic achievement.

## Data Availability

The raw data supporting the conclusions of this article will be made available by the authors upon request.
